# Chimeric Antigen Receptor T cell Therapy and the Immunosuppressive Tumor Microenvironment in Pediatric Sarcoma

**DOI:** 10.3390/cancers13184704

**Published:** 2021-09-20

**Authors:** Rachael L. Terry, Deborah Meyran, Emmy D. G. Fleuren, Chelsea Mayoh, Joe Zhu, Natacha Omer, David S. Ziegler, Michelle Haber, Phillip K. Darcy, Joseph A. Trapani, Paul J. Neeson, Paul G. Ekert

**Affiliations:** 1 Children’s Cancer Institute, Randwick 2031, Australia; rterry@ccia.org.au (R.L.T.); efleuren@ccia.org.au (E.D.G.F.); cmayoh@ccia.org.au (C.M.); d.ziegler@unsw.edu.au (D.S.Z.); mhaber@ccia.org.au (M.H.); 2 School of Women and Children’s Health, University of New South Wales, Randwick 2052, Australia; 3 Cancer Immunology Program, Peter MacCallum Cancer Centre, Melbourne 3000, Australia; Deborah.Meyran@petermac.org (D.M.); joe.zhu@petermac.org (J.Z.); phil.darcy@petermac.org (P.K.D.); joe.trapani@petermac.org (J.A.T.); paul.neeson@petermac.org (P.J.N.); 4 Sir Peter MacCallum Department of Oncology, University of Melbourne, Melbourne 3000, Australia; 5 Inserm, Université de Paris, U976 HIPI Unit, Institut de Recherche Saint-Louis, 75475 Paris, France; 6 Translational Innate Immunotherapy, University of Queensland Diamantina Institute (UQDI), Brisbane 4102, Australia; Natacha.Omer@health.qld.gov.au; 7 Oncology Services Group, Queensland Children Hospital, Brisbane 4101, Australia; 8 Kids Cancer Centre, Sydney Children’s Hospital, Randwick 2145, Australia; 9 Murdoch Children’s Research Institute, Royal Children’s Hospital, Melbourne 3052, Australia

**Keywords:** sarcoma, immunotherapy, CAR T cells

## Abstract

**Simple Summary:**

This review explores the current trials using cellular immunotherapies in pediatric sarcoma and describes examples of promising new CAR T targets in sarcoma that are in preclinical development. We provide insights into the ways in which the immunosuppressive tumor immune microenvironment can impact on CAR T cell therapy, highlighting specific mechanisms by which the tumor microenvironment may limit CAR T efficacy. Appreciation of these mechanisms may lead to rational combinations of immunotherapies, for example, the combination of CAR T cells with checkpoint inhibitor drugs. We also describe innovations in CAR T cell generation and combination therapies that may pave the way to better clinical outcomes for these patients.

**Abstract:**

Sarcomas are a diverse group of bone and soft tissue tumors that account for over 10% of childhood cancers. Outcomes are particularly poor for children with refractory, relapsed, or metastatic disease. Chimeric antigen receptor T (CAR T) cells are an exciting form of adoptive cell therapy that potentially offers new hope for these children. In early trials, promising outcomes have been achieved in some pediatric patients with sarcoma. However, many children do not derive benefit despite significant expression of the targeted tumor antigen. The success of CAR T cell therapy in sarcomas and other solid tumors is limited by the immunosuppressive tumor microenvironment (TME). In this review, we provide an update of the CAR T cell therapies that are currently being tested in pediatric sarcoma clinical trials, including those targeting tumors that express HER2, NY-ESO, GD2, EGFR, GPC3, B7-H3, and MAGE-A4. We also outline promising new CAR T cells that are in pre-clinical development. Finally, we discuss strategies that are being used to overcome tumor-mediated immunosuppression in solid tumors; these strategies have the potential to improve clinical outcomes of CAR T cell therapy for children with sarcoma.

## 1. Introduction

Sarcomas are rare cancers of the connective tissue that can arise anywhere in the body. There are over 100 different histological subtypes that occur in children, with osteosarcomas, Ewing’s sarcomas, and rhabdomyosarcomas being the most prevalent [1,2]. Together, they account for over 10% of childhood cancers [3]. This diversity of histology means that any one subtype of sarcoma is rare, and there has been little progress made towards new treatments in decades [4]. Current standard chemotherapies are toxic and have long-term side effects [5]. Furthermore, children with recurrent, relapsed, or metastatic disease have only a 20–30% chance of survival despite intensive chemotherapy, radiotherapy, and surgery [6].

There is now considerable interest in the potential for cellular immunotherapies to drive advances in treatment outcomes for children with sarcoma [7]. Chimeric antigen receptor (CAR) T cells are a promising type of adoptive cell therapy, where a patient’s own T cells are engineered to express a single-chain CAR, that typically comprises the extracellular binding domain of an antibody that recognizes a tumor cell surface antigen linked via a hydrophobic transmembrane domain to intracellular signaling motifs for T cell receptor (TCR) CD3zeta and a co-stimulatory molecule such as CD28 or 4-1BB. While normal T cell activation occurs when the TCR binds to peptide antigen presented on major histocompatibility complexes (MHC), CAR T cells can directly bind to tumor antigen, independently of MHC, and kill the tumor cells [8]. First generation CAR T cells contained the CD3zeta intracellular domain only and were largely ineffective, but second generation constructs that included a second, co-stimulatory signal, necessary for more effective T cell activation and persistence, have proven far more durable and effective in vivo and are now, by far, the most common format used clinically [9]. Third generation CAR T cells now encode two co-stimulatory domains, while the fourth generation, also known as “TRUCKS”, also incorporates a third (soluble) stimulatory signal—typically the local release of a pro-inflammatory cytokine such as IL-12, IL-15, IL-18, or IL-21 [10].

CAR T cell therapy has had, by far, its greatest success to date in treating hematological malignancies, particularly B cell cancers [11,12]. In 2017, the FDA approved CD19 CAR T cells to treat children with relapsed or refractory acute lymphoblastic leukemia and adolescents/adults with relapsed or refractory large B cell lymphoma [13]. However, despite extensive research, CAR T cell therapy has not yet had significant impact in sarcomas or, indeed, in any other solid tumor type. Of relevance to this review, only a small number of sarcoma patients have gained clinical benefit from a CAR T cell therapy despite apparently strong target antigen expression in some cases. One of the key contributors to this lack of efficacy is the intensely immunosuppressive tumor microenvironment (TME) inherent to most sarcomas [14]. In this review, we will discuss the CAR T cell therapies that are currently being tested in children with sarcoma, as well as new targets that are in pre-clinical development. We further discuss the strategies that are being investigated to overcome immunosuppression in the TME, which we propose have the potential to significantly improve CAR T cell therapy for children with sarcoma.

## 2. CAR T Cells in Clinical Trials for Pediatric Sarcomas

There are several clinical trials in progress testing CAR T cells in children with sarcoma, directed against different types of sarcoma antigen (Table 1). A number are membrane-bound receptor tyrosine kinases (RTKs), which represent one attractive class of CAR T targets in pediatric sarcomas, as many are recurrently overexpressed, and in some instances, are also driving oncogenes in these tumors [15,16]. Of all RTKs, CAR T cells directed against the human epidermal growth factor receptor 2 (HER2) have gained most clinical interest in pediatric sarcomas. HER2 is not considered a pediatric sarcoma driver. However, HER2 expression has been reported in several different subtypes, including osteosarcoma (~50%), Ewing’s sarcoma (16%), rhabdomyosarcoma (~33%), and desmoplastic small round cell tumor (DSRCT). The availability of HER2 CAR T cells developed for other, primarily adult malignancies, prompted trials in pediatric sarcomas [16,17,18]. In the first phase I/II trial testing HER2 CAR T in sarcomas, 19 patients with HER2-positive sarcomas were treated (NCT00902044). Although there were some concerns regarding safety after the death of an adult patient with colon cancer who received 1 × 10^10^ cells in another study [19], in this study, there were no evident toxicities. Promisingly, 3 out of 16 patients with recurrent or refractory osteosarcoma achieved stable disease (SD) for 12 to 15 weeks. Furthermore, after having their residual tumor removed, these three patients have remained in remission [17]. The single DSRCT patient in the study also achieved SD for 14 months. In the same trial, another group of patients was treated with HER2 CAR T cells after lymphodepletion, including five patients with osteosarcoma, three with rhabdomyosarcoma, and one patient each with Ewing’s sarcoma or synovial sarcoma. Excitingly, one of the five osteosarcoma patients who presented with lung metastases has so far maintained a CR for >32 months. A second patient with metastatic rhabdomyosarcoma showed a complete response (CR) for 12 months but subsequently relapsed. Upon receiving a further CAR T cell infusion, the patient achieved a CR that has been maintained for >15 months. A further three patients had SD after treatment. The 10 patients included had detectable CAR T at 6 weeks by qPCR [20]. A further report on this same trial described tumor remission and endogenous immune reactivity after administration of HER2 CAR T cells combined with pembrolizumab in a child with metastatic rhabdomyosarcoma, with a CR at 6 months [21]. If proven to be reproducible, such promising results in a heavily pre-treated cohort, with otherwise extreme prognosis, are quite exceptional and indicate that, for a small number of sarcoma patients, this treatment holds significant promise.

Clinical trials, in which patients with pediatric sarcoma were treated with CAR T cells. The table lists the trial registration number, CAR T cell antigen target, sarcoma subtype, clinical disease stage, patient age range, CAR T cell dose range, lymphodepletion regimen, and the trial status.

The epidermal growth factor (EGFR) RTK is also being explored as a CAR T target. EGFR is expressed in a range of sarcoma subtypes, including soft tissue sarcomas (~78%) and osteosarcomas (57%) [22,23]. A phase I clinical trial is currently recruiting children with EGFR-positive solid tumors (NCT03618381). Although it is still too early to draw conclusions, interim reports indicate that the therapy has promise as 23% of the trial subjects expressed EGFR on IHC, and over 60% of these EGFR-positive tumors were sarcomas, including osteosarcoma, Ewing’s sarcoma, rhabdomyosarcoma, and synovial sarcoma patients. No dose-limiting toxicities have been reported, so far, using the EGFR806-specific CAR alone, and its combination with a CD19-specific “driver” CAR is currently ongoing (NCT03618381). The main hypothesis is that CD19^+^ B cells serve in their normal role as antigen presenting cells to T cells and will therefore promote the expansion and persistence of the CAR T cells. However, a potential complication is that co-targeting CD19 is likely to deplete normal B cells.

Another CAR T target that has yielded promising clinical results in the treatment of children with sarcoma is NY-ESO-1, a cancer/testis antigen expressed in 47–80% of synovial sarcomas, 85% of myxoid liposarcomas, and 6–33% of conventional chondrosarcomas [24]. As a cell surface antigen, NY-ESO-1 can be targeted by CAR T cells, but also through adoptive immunotherapies that utilize TCR-transduced T cells that detect peptide antigen. This approach has been found to be promising for NY-ESO-1+ synovial sarcoma [25], as objective responses including tumor regression occurred in four out of six HLA-A*0201 patients with advanced disease treated with anti-NY-ESO-1 T cell receptor transduced T cells. Long-term follow-up confirmed objective responses in 11 out of 18 synovial sarcoma patients, including two long-lasting complete responses [26,27]. A subsequent multi-cohort phase I trial, with an updated protocol, has recently tested NY-ESO-1 CAR T cells in patients with synovial sarcomas following lymphodepletion. Patients that responded to the therapy for >3 months were treated with a second dose of NY-ESO CAR T cells. Promisingly, 1 patient achieved a CR after infusion, while 14 patients showed a PR. A further 25 patients showed SD after NY-ESO CAR T cell infusion, with 3 patients experiencing PD (NCT01343043) [28,29]. Of note, in early 2016, the FDA granted breakthrough therapy designation for the affinity enhanced T cell therapy, targeting NY-ESO-1 for advanced synovial sarcoma expressing NY-ESO-1 and certain specified HLA-A2 alleles—alleles expressed in ~79% (Peru) to only 12.7% (Myanmar) of the population, depending on the country studied [25]. A number of NY-ESO-1 directed T cell therapies have been initiated [30].

Similarly, the melanoma antigen gene (MAGE)-A4, a cancer/testis antigen expressed in many solid tumors, was targeted with specific peptide enhanced affinity receptor (SPEAR) T cells in phase I (NCT03132922) and phase II clinical trials (NCT04044768). Of 25 evaluable HLA-A2^+^ patients with inoperable or metastatic MAGE-A4+ synovial sarcoma or myxoid/round cell liposarcoma, 2 had a complete response, and 8 had a partial response. Comparable preliminary results were demonstrated in the prior phase I [31].

The ganglioside GD2 is also being explored as a CAR T cell target for children with sarcoma. GD2 expression has been reported in most osteosarcomas, as well as some Ewing’s sarcomas and rhabdomyosarcomas [32,33,34]. A phase I clinical trial testing dose escalation and expansion of a third generation GD2 CAR T cells was recently completed; however, the results are not yet available (NCT02107963). Three additional trials are active (NCT1953900), recruiting (NCT03635632), or not yet recruiting (NCT04539366). Although no clinical results have been published yet in pediatric sarcomas, GD2-directed CAR T cells, in combination with either a hepatocyte growth factor (HGF)-targeted neutralizing antibody or doxorubicin, were effective in preclinical Ewing’s sarcoma and osteosarcoma models [35,36]. This supports ongoing clinical trials in pediatric sarcoma directed against GD2 and highlights its potential for combination therapies.

Erythropoietin-producing hepatocellular receptor tyrosine class A2 (EphA2) is highly expressed in both osteosarcoma and Ewing’s, contrasting with its low expression in normal bone, and is reported to reveal targetable peptide epitopes in malignant tissue unavailable for binding in normal epithelial tissues. Anti-EphA2 CAR T intravenously infused in osteosarcoma, and Ewing’s sarcoma-bearing mice significantly improved their survival and eradicated metastatic deposits in liver and lungs in an aggressive metastatic osteosarcoma murine model [37]. These CAR T cells have not yet been tested in sarcoma patients but have demonstrated acceptable tolerance and transient clinical efficacy in recurrent glioblastoma [38].

B7-H3 (CD276) CAR T cells are also being tested for the treatment of pediatric sarcomas. B7-H3 is a member of the immunoglobulin superfamily, which may play a key role in suppressing T-cell responses to solid tumors. Expression of B7-H3 protein is described in a range of pediatric sarcomas, including osteosarcomas, rhabdomyosarcomas, Ewing’s sarcomas, synovial sarcomas, and desmoplastic small round cell tumors [39,40]. A phase I clinical trial is currently recruiting patients with solid tumors that express B7-H3 (NCT04483778). Several different CAR T cell constructs, targeting B7-H3, have been developed and showed tumor regression in preclinical mouse xenograft models osteosarcoma and Ewing’s sarcoma [41].

Glypican 3 (GPC3) is another promising target for CAR T cell therapy. GPC3 expression is reported in rhabdomyosarcoma and undifferentiated soft tissue sarcomas [39,40,42,43]. Two phase I clinical trials that are not yet recruiting, will be open to young patients with GPC3 tumors (NCT04377932; NCT04715191).

## 3. CAR T Cells in Pre-Clinical Development for Pediatric Sarcoma

In addition to the CAR T cell therapies in clinical trial, there are also several promising CAR T targets that have been tested against cell lines, in vitro and in animal models, of pediatric sarcomas (Table 2). CAR T cells have been developed to directly target the Receptor Tyrosine Kinases (RTK) signaling networks, activated by tyrosine kinase orphan-like receptor 1 (ROR1), 2 (ROR2), insulin-like growth factor 1 receptor (IGF-1R), vascular endothelial growth factor 2 (VEGF2), EPHA2 [37,44,45], and FGFR4 [42,43]. Other targets include the unique fusion protein, EWS-FLI-1, overexpressed antigens such as interleukin 11 receptor alpha subunit (IL11Rα), developmental/cancer testis antigens such as fetal acetylcholine receptor (fAChR), and costimulatory proteins such as NK cell activating receptor group2-member D (NKG2D) [44,45,46,47,48,49]. These CAR T cell approaches are in pre-clinical development but are yet to be trialed for safety or efficacy in patients.

Shown are the new CAR T cell targets being investigated in a range of pediatric sarcoma subtypes and the summary key data from each study.

Ewings Sarcoma-Friend leukemia integration 1 transcription factor (EWS-FLI-1), ephrin type-A receptor 2 (EPHA2), fetal Acetyl Choline Receptor (fAchR), Insulin-like Growth Factor-1 Receptor (IGF-1R), Interleukin-11 Receptor Alpha (IL11Ra), Natural Killer Group D (NKG2D), Pregnancy Associated Plasma Protein A (PAPPA), Platelet Derived Growth Factor Receptor Alpha (PDGFRA), Receptor tyrosine kinase-like orphan receptor 1 (ROR1), Vascular Endothelial Growth Factor Receptor 2 (VEGFR2).

## 4. Targeting the Immunosuppressive TME to Improve CAR T Cell Therapy for Pediatric Sarcomas

**Figure 1:**
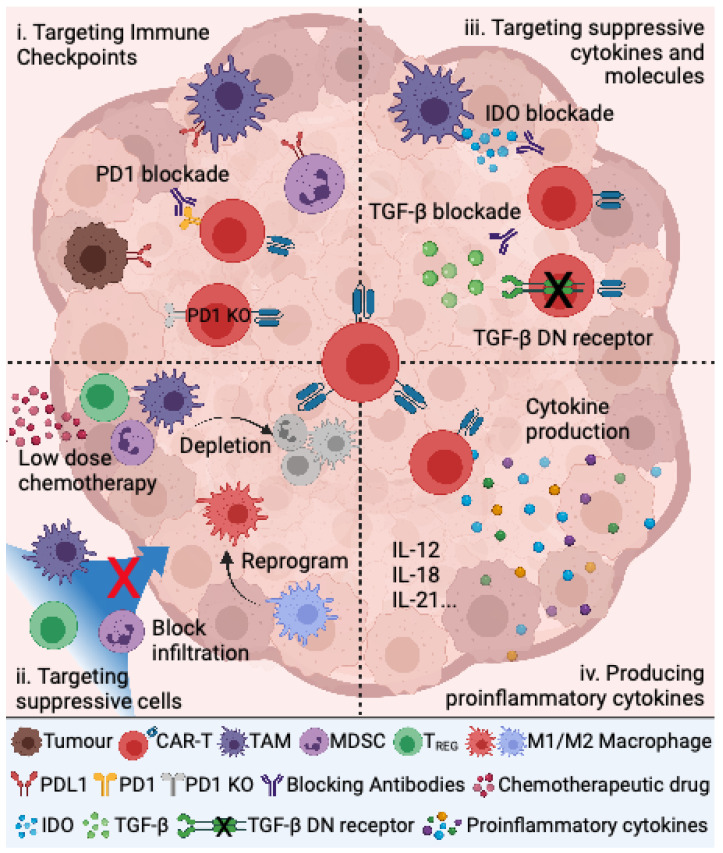
Strategies for addressing tumor-induced immune suppression in combination with CAR T cells to treat pediatric sarcoma.

In the limited trials to date, the majority of pediatric sarcoma patients fail to show any clinical response, notwithstanding the impressive responses in a small number of patients. The question is why do some patients respond, whilst others do not despite apparent target antigen expression? One significant contributing factor to this problem is the immunosuppressive nature of the solid tumor TME. This severely limits CAR T cell recruitment and infiltration into the tumor, CAR T cell survival, proliferation, and anti-tumor activity. In the following sections, we discuss potential approaches that involve modulation of the TME, or the CAR T cells themselves, to overcome mechanisms of immunosuppression in solid tumors that could be applied to improve the efficacy of CAR T cell therapy in sarcomas. These strategies are summarized in Figure 1.

### 4.1. Targeting Immune Checkpoints

Immune checkpoints are regulators of immune cell activation that suppress T cell-mediated responses in many cancer types [54]. PD-L1 is expressed by tumor cells and immunosuppressive immune cell populations, such as tumor-associated macrophages (TAMs) and myeloid-derived suppressor cells (MDSCs); PD-L1 inhibits T cell activation by binding to PD-1 expressed by T cells [55]. Combination therapy with anti-PD-1 can boost the efficacy of CAR T cell therapy [56] by limiting tumor-mediated CAR T cell exhaustion [57]. PD-1 blockade in combination with HER2 CAR T cell therapy has so far achieved promising results in one child with rhabdomyosarcoma. This patient was first successfully treated with HER2 CAR T cells alone, but after disease relapse at 6 months off therapy, additional HER2 CAR T infusions were given together with pembrolizumab. This patient is now 20 months post T cell infusions with no detectable disease [21]. Another approach to mitigating the PD-1/PD-L1 axis is to knockout PD-1 expression by CAR T cells. This strategy is currently being tested in over 20 clinical trials in adult cancers, including those of the breast, lung, and prostate [58]. The potential of this is exemplified by several studies. Rupp et al. demonstrated, using CD19^+^ PD-L1^+^ K562 cells, that PD-1 disrupted CAR T cells significantly enhanced anti-tumor efficacy in vitro and in vivo (tumor clearance rate of 17% for conventional anti-CD19 CAR T cells increased to 100% in animals receiving PD-1 edited CAR T cells at 28 days post tumor implant) [58,59]. Hu et al. showed that PD-1 disruption improved the in vitro and in vivo anti-tumor activity of CD133-specific CAR T cells in an orthotopic mouse model of glioma, without apparent toxicity [60].

Other approaches include the use of dominant-negative PD-1 receptor CAR T cells and triple inhibitory receptor CAR T cells that downregulate PD-1, as well as two other immune checkpoint molecules (TIM-3 and LAG-3). Genetic blockade of these three checkpoint inhibitory receptors resulted in high expression of CD56 on CAR T cells, and enhanced inhibition of tumor growth [61]. A high expression of PD-L1 was detected in alveolar rhabdomyosarcoma (86%), Ewing’s sarcoma (57%), embryonal rhabdomyosarcoma (50%), and osteosarcoma (47%) [62]. In addition, there are a multitude of immune checkpoints beyond the PD-1/PD-L1 axis [63], which may be more relevant to target in different subsets of pediatric sarcomas, in conjunction with CAR T cell therapy. For instance, a higher expression of CTL-4 in circulating CD4^+^ and CD8^+^ T cells was detected in children affected by aggressive sarcomas [64]. Recently, the largest screen of B7-H3 expression in pediatric tumors showed a high and homogeneous expression in rhabdomyosarcoma and Ewing’s sarcoma [41,65]. In advanced osteosarcoma, TIGIT^+^ T cells are highly present supporting the potential clinical application of TIGIT blockade in this tumor [66]. Although further research is needed to pinpoint the most appropriate targets, the examples of superior efficacy of combined PD-1 blockade, together with CAR T cell therapy, indicates the potential promise of CAR T cell modifications. Extending this to other immune checkpoint targets more specific to pediatric sarcomas could improve clinical response rates.

### 4.2. Targeting Suppressive Immune Cells

The TME is a heterogenous network of immune cell populations, including immune cell subsets that support tumor growth, survival, and suppress cytotoxic T cell responses, such as tumor-associated macrophages (TAMs), myeloid-derived suppressor cells (MDSCs), and regulatory T cells (T_reg_) [67]. Several approaches that inhibit these cell types in the TME are being tested. Low dose chemotherapy, with agents such as 5-fluorouracil, paclitaxel, cisplatin, and gemcitabine deplete MDSCs [68] and could potentially be used in combination with CAR T cell therapy to boost anti-tumor responses. Low dose chemotherapy was also found to deplete T_reg_ cells [69]. Indeed, low dose chemotherapy added to the conditioning regimen has been shown to boost CAR T cell activity in a lung tumor model by activating tumor macrophages to express T-cell recruiting chemokines [70].

Blockade of the chemokines that TAMs, MDSCs, and T_reg_ cells use to traffic to the tumor is another approach to deplete these immunosuppressive subtypes from the TME. Antibodies that block the CCL2/CCR2 axis have shown promising results in depleting myeloid cells from the TME in several pre-clinical models [71]. The CXCR4/CXCL12 pathway is another target for inhibition that could be combined with CAR T cell therapy to reduce the number of immunosuppressive myeloid cells within the TME [72]. The cytokine Colony Stimulating Factor 1 (CSF-1) is an important regulator of myeloid cell recruitment. Blockade of the receptor of this cytokine (CSF1R) has been shown not only to reduce the recruitment of myeloid cells into the tumor but to also preferentially promote differentiation into an M1 pro-inflammatory phenotype, as opposed to an M2 anti-inflammatory phenotype [73]. The chemokine receptor CCR4 is highly expressed on T regulatory cells and blockade of the CCR4/CCL22 axis could be used to reduce the number of these cells in the TME [74]. Several tyrosine kinase inhibitors have also been found to reduce MDSC [75,76] and regulatory T cells [77] in the TME and could, potentially, be used in conjunction with CAR T cell therapy in sarcoma.

Low dose radiotherapy has been shown to reprogram TAM into M1 pro-inflammatory macrophages in several studies [78,79] and to reduce the numbers of T regs in the TME [80]. There are also some promising results using toll-like receptor agonists to promote M1 macrophage polarization in the TME [72]. RNA-based therapies are emerging that promote M1 macrophage differentiation, by downregulating the expression of M2 genes [81,82]. Targeting the BTK–PI3Kγ pathway has been shown to reprogram TAMs into M1 macrophages in several studies [83,84,85] and has the potential to be combined with CAR T cell therapy to improve their efficacy. However, first generation BTK inhibitors suppress NK-cell cytotoxicity so could potentially inhibit CAR T cell function. This was not observed with second generation BTK inhibitors, highlighting the need for careful consideration of the most appropriate combination therapies when using this class of drug with CAR T therapy [86].

### 4.3. Targeting Immunosuppressive Cytokines and Other Molecules

Tumor cells and immunosuppressive immune cells in the TME produce a range of anti-inflammatory cytokines that suppress CAR T cell proliferation and activity. Blockade of transforming growth factor beta (TGFβ) signaling has been shown to reduce tumor growth and promote T cell-driven anti-tumor responses in vivo [87]. Another approach is to engineer CAR T cells with a TGFβ dominant negative receptor to mitigate the suppressive effect of TGFβ signaling on CAR T cell responses. This has been shown to increase the efficacy of these CAR T cells in models of prostate cancer [88]. Indoleamine 2,3, dioxygenase (IDO1) is another anti-inflammatory molecule produced by TAM and other immunosuppressive immune cells in the TME, which suppresses T cell activation and function [89]. Blockade of IDO significantly improved the efficacy of CAR T therapy in a pre-clinical model of colon cancer [90].

### 4.4. Production of Proinflammatory Cytokines

Another way to boost the efficacy of CAR T cells is to directly engineer them to express pro-inflammatory cytokines (TRUCKs or ARMOURED CARs) [91]. IL-12 enhances T cell cytotoxicity and induces the production of IFNγ driving the Th1 response. IFNγ in turn activates monocytes and macrophages to further increase IL-12 production [92]. CAR T cells producing IL-12 have shown increased anti-tumor efficacy [93]. Furthermore, IL-23 antagonists improve the IL-12: IL-13 ratio in favor of IL-12, with the potential to be used in combination with CAR T cells [94]. IL-18 promotes both Th1 and Th2 responses and, in conjunction with IFNγ production, recruits and activates innate immune cells and enhances further IFNγ production [95]. CAR T cells that constitutively express IL-18 have also shown increased efficacy in a model of melanoma [96]. IL-21 is a pleiotropic cytokine that is produced by T cell subsets and enhances the survival and anti-tumor activity of CD8^+^ T cells [97]. Indeed, CAR T cells, producing IL-21, have shown increased efficacy against tumor cells in vivo [98].

## 5. Understanding the Immunosuppressive TME Is Key to Improving CAR T Cell Therapy

There are many different methods that can be deployed to target the immunosuppressive TME of solid tumors. However, it is currently unclear which method should be applied to which subsets of pediatric sarcomas. Indeed, the astonishing diversity of histological and molecular subtypes of sarcoma might suggest that there will be many immunosuppressive pathways operating in these entities. A detailed characterization of the immune TME of pediatric sarcomas will provide the necessary insights into the immunosuppressive mechanisms that should be targeted in different sarcoma subtypes. This detailed characterization of the immune TME is mostly lacking [99]. Indeed, even at a basic level, there is little data to indicate whether subsets of pediatric sarcomas are immune-inflamed and may respond to CAR T cell therapy, as well as which subsets would require specific co-therapies to boost CAR T cell efficacy. A better understanding of the TME of different sarcoma subtypes will undoubtedly allow for more specific targeting of the immunosuppressive mechanisms that limit CAR T cell recruitment and migration into the tumor, as well as their proliferation and persistence [100].

Solid tumors can be classified into three subtypes based on their immune microenvironments. Immune-inflamed “hot” tumors have significant numbers of CD8^+^ T cells in the parenchyma and express pro-inflammatory cytokines, chemokines, and immune checkpoints [101]. These patients could be anticipated to be the best responders to CAR T cell therapy as they already permit endogenous T cell infiltration into the TME [102]. As they also express immune checkpoint molecules, they may also benefit from co-therapy with immune checkpoint inhibitors.

Immune-altered “intermediate” tumors are characterized by the restriction of T cell infiltrate to the surrounding stroma, with production of chemokines and tumor-derived inhibitory factors, such as TGFβ [101]. To improve CAR T cell therapy, these tumors could potentially benefit from co-therapies that target immunosuppressive cytokines, such as TGFβ inhibitors, or by treatment with fourth generation CAR T cells that express inflammatory cytokines.

Immune desert “cold” tumors completely lack CD8^+^ T cells and are populated with immunosuppressive immune cell populations such as TAMs, MDSCs, and regulatory T cells [101]. The cytokine milieu in these tumors is that of immune suppression and tolerance. These tumors would be expected to be the poorest responders to CAR T cell therapy as the same mechanisms that exclude endogenous T cell infiltrate into the TME would be expected to limit CAR T cell recruitment [102]. These tumors present the most challenging TME for immunotherapies. Co-treatment with low dose chemotherapy, radiotherapy, or targeted therapy, which significantly modify the TME and reduce immunosuppressive immune cell populations, may have the greatest impact on efficacy. Cisplatin is one of the main chemotherapies used in standard protocols to treat osteosarcoma [103]. Similarly, radiotherapy is standard of care for both rhabdomyosarcoma and Ewing’s sarcoma [104]. CAR T cell therapy could be combined with standard protocols to treat certain subtypes of refractory sarcoma.

There are also intrinsic features of CAR T cells which may limit their efficacy, but which may, nonetheless, be amenable to intervention. This has been reviewed elsewhere [105]. However, some interesting lessons from the application of CAR T therapies in leukemia may be relevant to sarcoma immunotherapy. One recent line of investigation is the capacity of bromodomain inhibitors to maintain expression of the CAR and prolong CAR T cell persistence [106,107]. Epigenetic mechanisms appear to play a significant role in diminishing CAR expression over time, perhaps a result of the epigenetic profile of the parent T-cells from which the CAR T cells in the final vaccine product are derived [106], as well as the non-random sites of lentiviral integration during CAR T cell manufacture [107,108]. Treatment of CAR T cells, during the in vitro expansion phase with the BET inhibitor drug JQ1, can maintain a memory-cell phenotype, prolonging expression of the CAR and persistence of the CAR T cells. The wider application of CAR T therapy in hematological malignancies also raises questions about the possibility of high-quality donor cells that may be derived from sources other than the individual patient (so-called non-autologous, “off-the-shelf” CAR T cells). Here, the balance is between using T cells, which are more readily available, and the risks of eliciting graft versus host disease. At least one study has shown that deletion of the endogenous TCR in such CAR T cells greatly reduces their alloreactivity, but at some cost to efficacy [109].

## 6. Conclusions

CAR T cell therapy harnesses a patient’s own immune system to recognize and kill cancer cells. It has the potential to improve clinical outcomes for children with sarcomas that are resistant to standard therapies or who have relapsed. There are currently several clinical trials open to children with tumors that express HER2, NY-ESO, GD2, B7-H3, EGFR, and GPC3, and some promising results have already been reported, notably using HER2 CAR T cells, in which complete responses have been achieved in patients with osteosarcoma and rhabdomyosarcoma [20] and with NY-ESO-1+ synovial sarcomas treated with NY-ESO CAR T cells [28,29]. However, there are many other promising options for the future development of cell-based immunotherapies, including CAR T cells directed against new, promising targets such as EPHA2. EPHA 2 CAR T cells have very recently entered a first-in-human clinical trial in adult glioblastoma [38]. Although optimization will be required, these exciting developments pave the road towards further testing of EPHA2 CAR T cell-directed therapies in childhood sarcoma patients, ideally in combination with strategies that tackle the immunosuppressive barriers in these tumors.

One of the main limitations in CAR T trials, in children with solid tumors, is the recruitment of patients based solely on tumor antigen expression rather than on the subtype of their cancer. As a result, patients with different types of cancer may receive the same CAR T product without considering the features of their specific TME, which is a key contributor to failed CAR T cell responses [110]. At this time, we do not routinely screen for sarcomas that are immune-inflamed and may permit CAR T cell infiltration into the TME or distinguish them from those that are not inflamed and are likely to exclude or lack CAR T cell infiltrates in the TME. However, tackling this immunosuppressive barrier, for example, with a PD-1 blockade, can increase efficacy of CAR T cell mediated therapy. This clearly mandates a deeper investigation into the unique TME of pediatric sarcomas. Identification of checkpoint inhibitors, or other TME components specific to pediatric sarcomas, holds the promise to increase efficacy of CAR T cells and tailored CAR T cell-focused combination therapies.

Pediatric sarcomas present several obstacles for CAR T cell efficacy. These obstacles can be divided into four main CAR T cell combination treatment strategies by (1) targeting immune checkpoint molecules and ligands, (2) targeting immune suppressor cells in the tumor microenvironment, (3) targeting suppressive cytokines and molecules, and (4) producing pro-inflammatory cytokines.

## Figures and Tables

**Table 1:** CAR T cell clinical trials in pediatric sarcoma.

Trial Number	Target Antigen	Sarcoma Subtype	Phase	Age (Years)	Dose	Lymphodepletion	Status
NCT00902044	HER2	All sarcoma	I/II	All	1 × 10^4^–1 × 10^8^/m^2^	Fludarabine, Cyclophosphamide	Completed, with results
NCT00889954	HER2	All cancers	I	3+	1 × 10^4^–1 × 10^8^/m^2^	No	Completed
NCT01343043	NY-ESO-1	Synovial	I	4+	>40 kg: 1 × 10^9^–6 × 10^9^ <40 kg: 0.025 × 10^9^ cells/kg	Fludarabine, Cyclophosphamide	Completed, with results
NCT03638206	NY-ESO-1	All cancers	I/II	4–70	Not specified	Cyclophosphamide or Fludarabine	Recruiting
NCT02107963	GD2	All	I	<36	1 × 10^5^–1 × 10^7^ cells/kg	Cyclophosphamide	Completed
NCT1953900	GD2	Osteosarcoma	I		1 × 10^6^–1 × 10^9^	Fludarabine, Cyclophosphamide	Active
NCT03635632	GD2	All	I	1–74	1 × 10^7^–1 × 10^8^	Fludarabine, Cyclophosphamide	Recruiting
NCT04539366	GD2	Osteosarcoma	I	<36	Not specified	Fludarabine, Cyclophosphamide	Not yet recruiting
NCT0372106 8	GD2	Osteosarcoma	I	1.5–18	0.5 × 10^6^–1.5 × 10^6^	Fludarabine, Cyclophosphamide	Recruiting
NCT04483778	B7-H3	All	I	0–27	Not specified	Not specified	Recruiting
NCT04897321	B7-H3	All	I	0–21	Not specified	Fludarabine, Cyclophosphamide	Not yet recruiting
NCT04864821	B7-H3	All	I	1–70	Not specified	Not specified	Not yet recruiting
NCT03618381	EGFR	All	I	1–26	Not specified	Not specified	Recruiting
NCT04377932	GPC3	All	I	1–21	3 × 10^7^–3 × 10^8^/m^2^	Fludarabine, Cyclophosphamide	Not yet recruiting
NCT04715191	GPC3	All	I	1–21	1 × 10^8^–1 × 10^9^/m^2^	Fludarabine, Cyclophosphamide	Not yet recruiting

**Table 2:** New CAR T cell targets for pediatric sarcoma currently under development in pre-clinical models.

Target Antigen	Sarcoma Subtype	In Vitro and In Vivo Responses	Reference
EWS-FLI-1	Ewing’s sarcoma	▪ Cytotoxicity against 8/11 Ewing’s sarcoma cell lines Increased survival of mice bearing Ewing’s sarcoma cell line TC-71	[44]
EPHA2	Osteosarcoma and Ewing’s sarcoma	▪ Cytotoxicity against osteosarcoma and Ewing’s sarcoma cell lines ▪ Produced high levels of IFN-γ and IL-2 ▪ Tumor regression in mice bearing osteosarcoma cell line 143B and Ewing’s sarcoma cell line A673	[37]
fAchR	Rhabdomyosarcoma	▪ Production of IFN and granzyme B in co-cultures with rhabdomyosarcoma cell line TE-671 cells transfected with fetal AChr ▪ Cytotoxicity against rhabdomyosarcoma cell line TE-671 cells transfected with fetal AChR	[45]
fAchR	Rhabdomyosarcoma	▪ Delayed tumor growth in mice bearing rhabdomyosarcoma cell line RD6 ▪ Tumor growth further delayed by co-treatment with the surviving inhibitor Shepherdin	[48]
FGFR1		▪ CAR T developed using specific single domain antibody ▪ Cytotoxicity against FGFR4-expressing rhabdomyosarcoma cell lines (RH4) in vitro ▪ Specificity demonstrated by FGFR4 gene-deletion	[42]
IGF-1R	Rhabdomyosarcoma, Osteosarcoma, Ewing’s sarcoma, Fibrosarcoma	▪ Cytotoxicity against a large panel of rhabdomyosarcoma, Ewing’s sarcoma, fibrosarcoma and osteosarcoma cell lines ▪ Produced high levels of Th1 cytokines (IFN-γ and TNF-α) ▪ Delayed tumor growth in mice bearing osteosarcoma cell line SaOS2	[47]
IL11Ra	Osteosarcoma	▪ Cytotoxicity against 4 osteosarcoma cell lines in vitro ▪ Reduced lung metastases in mice bearing osteosarcoma cell line KRIB	[46]
NKG2D	Ewing’s sarcoma	▪ Cytotoxicity against Ewing’s sarcoma cell lines	[50]
PAPPA	Ewing’s sarcoma	▪ Cytotoxicity against A673 cell line ▪ Delayed tumor growth in mice bearing Ewing’s sarcoma cell line A673	[51]
PDGFRA	Rhabdomyosarcoma	▪ Cytotoxicity against rhabdomyosarcoma cell line RD ▪ Produced high levels of pro-inflammatory cytokines (IL-2, IL-13, GM-CSF, MIP-1α, IFN-γ, TNF-α) ▪ Delayed tumor growth and increased survival in mice bearing rhabdomyosarcoma cell line RD	[52]
ROR1	Osteosarcoma	▪ Cytotoxicity against osteosarcoma cell line SaOS2 ▪ Produced high levels of Th1 cytokines (IFN-γ and TNF-α) ▪ Delayed tumor growth in mice bearing SaOS2	[47]
VEGFR2	Ewing sarcoma	▪ VEGFR2 expressed on tumor-associated blood vessels ▪ Specific cytotoxicity of VEGFR2-expressing targets	[53]
